# Advisory Committee on Immunization Practices Recommended Immunization Schedule for Children and Adolescents Aged 18 Years or Younger — United States, 2023

**DOI:** 10.15585/mmwr.mm7206a1

**Published:** 2023-02-10

**Authors:** A. Patricia Wodi, Neil Murthy, Veronica McNally, Sybil Cineas, Kevin Ault

**Affiliations:** ^1^Immunization Services Division, National Center for Immunization and Respiratory Diseases, CDC; ^2^Franny Strong Foundation, West Bloomfield, Michigan; ^3^The Warren Alpert Medical School of Brown University, Providence, Rhode Island; ^4^Western Michigan University Homer Stryker MD School of Medicine, Kalamazoo, Michigan.

At its October 2022 meeting, the Advisory Committee on Immunization Practices* (ACIP) approved the Recommended Child and Adolescent Immunization Schedule for Ages 18 Years or Younger, United States, 2023. The 2023 child and adolescent immunization schedule, available on the CDC immunization schedule website (https://www.cdc.gov/vaccines/schedules), summarizes ACIP recommendations, including several changes from the 2022 immunization schedule^†^ on the cover page, tables, notes, and appendix. Health care providers are advised to use the tables, notes, and appendix together to determine recommended vaccinations for patient populations. This immunization schedule is recommended by ACIP (https://www.cdc.gov/vaccines/acip) and approved by CDC (https://www.cdc.gov), the American Academy of Pediatrics (https://www.aap.org), the American Academy of Family Physicians (https://www.aafp.org), the American College of Obstetricians and Gynecologists (http://www.acog.org), the American College of Nurse-Midwives (https://www.midwife.org), the American Academy of Physician Associates (https://www.aapa.org), and the National Association of Pediatric Nurse Practitioners (https://www.napnap.org).

ACIP’s recommendations for the use of each vaccine are developed after in-depth reviews of vaccine-related data, including the epidemiology and societal impacts of the vaccine-preventable disease, vaccine efficacy and effectiveness, vaccine safety, quality of evidence, feasibility of program implementation, and economic analyses of immunization policy (*1*). The child and adolescent immunization schedule is published annually to consolidate and summarize updates to ACIP recommendations on vaccination of children and adolescents and to assist health care providers in implementing current ACIP recommendations. The use of vaccine trade names in this report and in the child and adolescent immunization schedule is for identification purposes only and does not imply endorsement by ACIP or CDC.

For further guidance on the use of each vaccine, including any changes that might occur after annual publication of the 2023 child and adolescent immunization schedule, health care providers are referred to the respective ACIP vaccine recommendations at https://www.cdc.gov/vaccines/hcp/acip-recs. If errors or omissions are discovered within the schedule, CDC will post revised versions on the CDC immunization schedule website.^§^ Printable versions of the 2023 child and adolescent immunization schedule and instructions for ordering hard copies of the schedule are available on the immunization schedule website (https://www.cdc.gov/vaccines/schedules/).

## Changes in the 2023 Child and Adolescent Immunization Schedule

Vaccine-specific changes in the 2023 immunization schedule for children and adolescents aged ≤18 years include new or updated ACIP recommendations for influenza vaccine (*2*), pneumococcal conjugate vaccine (*3*), measles, mumps, and rubella vaccine (MMR) (*4*), and COVID-19 vaccine (https://www.cdc.gov/vaccines/hcp/acip-recs/vacc-specific/covid-19.html), which have been added to the Tables and to the Notes sections. Changes also include clarification of the recommendations for dengue vaccine, hepatitis A vaccine (HepA), hepatitis B vaccine (HepB), human papillomavirus vaccine (HPV), meningococcal serogroups A, C, W, Y vaccine (MenACWY), meningococcal serogroup B vaccine (MenB), inactivated poliovirus vaccine (IPV), and varicella vaccine.

### Cover page

• COVID-19 vaccines, 15-valent pneumococcal conjugate vaccine (PCV15), and a newly licensed MMR (Priorix) have all been added to the table of vaccine abbreviations and trade names.

### Table 1 (Routine Immunization Schedule)

• **COVID-19 row:** A new row has been added with the columns for age 6 months–18 years highlighted in yellow to indicate the recommended age for COVID-19 vaccination. The overlying text “2- or 3-dose primary series and booster (See Notes)” has also been added.

• **Pneumococcal conjugate row:** PCV15 has been added.

• **IPV row:** The overlying text “See Notes” has been added to the column for persons aged 17–18 years prompting health care providers to review the Notes section for additional information for persons aged 18 years.

### Table 2 (Catch-up Immunization Schedule)

• **Pneumococcal conjugate row:** Language for the minimum interval between doses 3 and 4 has been revised to clarify when a fourth dose is indicated. The text now reads “This dose is only necessary for children aged 12–59 months regardless of risk, or aged 60–71 months with any risk, who received 3 doses before age 12 months.”

### Table 3 (Immunization by Medical Indication Schedule)

• **COVID-19 row:** A new row was added to summarize COVID-19 vaccination recommendations by medical conditions or other indications. The overlying text “See Notes” has been added to both HIV infection and immunocompromised status (excluding HIV infection) columns prompting providers to review specific recommendations for these populations.

### Vaccine Notes

The notes for each vaccine are presented in alphabetical order. Edits have been made throughout the Notes section to harmonize language between the child and adolescent immunization schedule and the adult immunization schedule to the greatest extent possible.

• **Additional information:** The text for injury compensation was revised to include the Countermeasures Injury Compensation Program for COVID-19 vaccines.

• **COVID-19:** A new section was added to provide additional details on the use of COVID-19 vaccines. The routine vaccination section describes the recommendations for primary series in the general population, and the special situations section describes the recommendations for primary series in persons who are moderately or severely immunocompromised. For booster dose vaccination in all populations, and guidance for Janssen (Johnson & Johnson) COVID-19 vaccine recipients, hyperlinks are included referring health care providers to the latest guidance. In addition, hyperlinks to the current COVID-19 vaccination schedules, use of COVID-19 preexposure prophylaxis in persons who are moderately or severely immunocompromised, as well as Emergency Use Authorization indications for COVID-19 vaccines, have been added.

• **Dengue:** A new bullet was added to clarify that dengue vaccine should not be administered to children traveling to or visiting endemic dengue areas.

• **HepB**: The language in the routine vaccination section was revised to highlight the recommendations for infants born to mothers who have received positive test results for hepatitis B surface antigen (HBsAg), or whose HBsAg status is unknown. In addition, the catch-up vaccination section was updated to include Heplisav-B and PreHevbrio vaccines for persons aged 18 years.

• **Influenza:** The note has been updated to reflect the recommendations for the 2022–23 influenza season. Language was added to the “Special situations” section to clarify that live attenuated influenza vaccine should not be administered to close contacts of immunosuppressed persons who require a protected environment. In addition, the language for persons with egg allergy with symptoms other than hives was moved from the appendix to the “Special situations” section.

• **MMR:** The “Special situations” section was updated to include recommendations for additional MMR doses in a mumps outbreak setting.

• **MenACWY:** Language clarifying that the newly licensed Menveo one-vial (all liquid) formulation should not be administered before age 10 years was added.

• **MenB:** The “Special situations” section was updated to include the recommendations for situations in which the second or third dose of Trumenba is administered earlier or later than the recommended minimum interval. If the second dose is administered ≥6 months after the first dose, then the third dose is not needed. If the third dose is administered earlier than 4 months after the second dose, a fourth dose should be administered ≥4 months after the third dose.

• **Pneumococcal:** The routine vaccination, catch-up vaccination, and “Special situations” sections have been updated with the recommendations for use of PCV15. In addition, language was added stating that 13-valent pneumococcal conjugate vaccine (PCV13) and PCV15 can be used interchangeably in both healthy children and those with any risk for invasive pneumococcal disease. In addition, a hyperlink to the CDC app that can be used to determine a patient’s pneumococcal vaccination needs has been included.

• **Poliovirus:** A new “Special situations” section was created to describe the use of IPV in persons aged 18 years who are at increased risk for exposure to polioviruses.

### Appendix (Contraindications and Precautions)

• The column header was changed from “Contraindications” to “Contraindicated or Not recommended.”

• **Influenza (egg-based) row:** In the precautions for egg-based inactivated and live attenuated vaccines, the language for persons with egg allergy with symptoms other than hives has been moved to the Notes section.

• **Dengue row:** Language was added stating that lack of laboratory confirmation of previous dengue virus infection is a contraindication.

• **HepB row:** Language was added to the contraindicated or not recommended column stating that Heplisav-B and PreHevbrio are not recommended during pregnancy; other HepB products should be used if vaccination is indicated. A footnote providing information on the pregnancy exposure registries for persons who were inadvertently vaccinated with Heplisav-B or PreHevbrio while pregnant was added.

• **HPV row:** Language was added to the contraindicated or not recommended column stating that HPV is not recommended during pregnancy.

• **MMR row:** Measles, mumps, rubella, and varicella virus vaccine (MMRV) was added. In addition, language was added to the precautions stating that a personal or family history of seizure of any etiology is a precaution for using MMRV.

• **Varicella row:** Language was added stating that if MMRV is used, the precautions for MMR/MMRV should be reviewed.

## Additional Information

The Recommended Child and Adolescent Immunization Schedule, United States, 2023 is available at https://www.cdc.gov/vaccines/schedules/hcp/imz/child-adolescent.html. The full ACIP recommendations for each vaccine are also available at https://www.cdc.gov/vaccines/hcp/acip-recs. All vaccines identified in Tables 1, 2, and 3 (except DTaP, rotavirus, and PCV13) also appear in the Recommended Adult Immunization Schedule for Ages 19 Years or Older, United States, 2023, available at https://www.cdc.gov/vaccines/schedules/hcp/imz/adult.html. The notes and appendix for vaccines that appear in both the child and adolescent immunization schedule and the adult immunization schedule have been harmonized to the greatest extent possible.

## Footnotes

* Recommendations for routine use of vaccines in children and adolescents are developed by ACIP, a federal advisory committee chartered to provide expert external advice and guidance to the CDC director on use of vaccines and related agents for the control of vaccine-preventable diseases in the civilian population of the United States. Recommendations for routine use of vaccines in children and adolescents are harmonized to the greatest extent possible with recommendations made by the American Academy of Pediatrics, the American Academy of Family Physicians, the American College of Obstetricians and Gynecologists, the American College of Nurse-Midwives, the American Academy of Physician Associates, and the National Association of Pediatric Nurse Practitioners. ACIP recommendations approved by the CDC director become agency guidelines on the date published in the *Morbidity and Mortality Weekly Report*. Additional information about ACIP is available at https://www.cdc.gov/vaccines/acip.

† Past immunization schedules are available at https://www.cdc.gov/vaccines/schedules/hcp/schedule-related-resources.html#accordion-2-collapse-3.

§ CDC encourages organizations to use syndication as a more reliable method for displaying the most current and accurate immunization schedules on an organization's website rather than copying these schedules to their websites. Use of content syndication requires a one-time step that ensures an organization’s website displays current schedules as soon as they are published or revised; instructions for syndication code are available on CDC’s website https://www.cdc.gov/vaccines/schedules/resource-library/syndicate.html. CDC also offers technical assistance for implementing this form of content syndication (requests can be e-mailed to ncirdwebteam@cdc.gov).
